# Primary total knee arthroplasty for complex supracondylar femoral fractures in patients with knee arthritis: A retrospective study of a patient cohort

**DOI:** 10.1097/MD.0000000000012700

**Published:** 2018-10-05

**Authors:** Wei Wang, Kun Yang, Pei Yang, Dandan Song, Chunsheng Wang, Jinhui Song, Xiaohui Li, Kunzheng Wang

**Affiliations:** Department of Orthopedics, The Second Affiliated Hospital of Xi’an Jiaotong University, Xi’an, China.

**Keywords:** arthritis, arthroplasty, knee, stemmed femoral component, supracondylar fracture

## Abstract

When elder arthritis patients suffered from complex supracondylar femoral fractures, their joints condition and general health condition elevate the difficulties in operation and post-surgical recovery.

Here, we aimed to simplify the operation procedure by using one-step Total Knee Arthroplasty (TKA) with a stemmed femoral implant. We also investigated if this method could improve the patients’ experience after the operation. The surgery including femoral osteotomy and implantation was performed on all fourteen patients by the same orthopedic specialist. The patients’ hospitalization time was recorded. The recovery of knee function and patient satisfaction was evaluated by a systematic follow-up with average time 38 months, up to 5 5 years, using Hospital for Special Surgery (HSS) knee scores, the range of motion (ROM), anteroposterior and lateral radiography, and Visual Analog Scale (VAS) scores. The average of hospitalization days was 16 days. No angular deformity, malunion, or shortening were found at radiography.

The average ROM was 105.2° at the end of the follow-up period. The knees in all the patients show adequate stability. All patients had returned to their former daily activities. Seventeen out of 24 patients were satisfied with the outcome of the surgery.

The usage of TKA with a stemmed femoral implant is a reasonable method for elderly patients suffering from supracondylar femoral fractures and concomitant knee arthritis.

## 1 Introduction

The age of supracondylar femoral fracture patients displays a bimodal distribution.^[1]^ Supracondylar femoral fracture in young patients is usually caused by high energy trauma, including traffic incidents, falls from heights and sports injuries. The commonly applied treatment includes anatomical reduction and internal fixation.^[2]^ On the other hand, elderly patients, particular females, are more venerable to even mild traumatic injuries, such as a slight slip or fall.^[1,3]^ It is difficult to treat these patients who are suffering from osteoporosis and age-associated general health conditions. Many elderly patients suffer from knee arthritis, joint deformity, and other arthritic changes. This represents a therapeutic challenge.^[4–7]^

The most frequently applied operative treatment for supracondylar femoral fracture includes 95-degree angled blade plates, condylar screws, and retrograde nailing.^[8–10]^ However, these methods do not solve the issue of preexisting arthritis which may comprise the outcome of treatment. Therefore, primary total knee arthroplasty (TKA) was suggested for treating supracondylar femoral fractures in patients suffering from knee arthritis. One of the most commonly performed TKA procedures is a 2-stage procedure, consisting of fractures repair, followed by TKA operation after bone reunion. However, this procedure usually leads to decreased knee range of motion (ROM) in many cases because of arthrofibrosis,^[11,12]^ and the alteration of femur axis.^[13]^ This procedure, therefore, makes it difficult to perform subsequent TKA. Moreover, as both steps are complicated surgeries requiring different large skin incisions, this procedure may result in scar contracture of the skin or skin necrosis. The other option is primary TKA with customized components, such as a constrained prosthesis.^[14,15]^ This type of prosthesis requires a large intercondylar cut, which may decrease fracture stability in case of juxta-articular fractures. It is the aim of our study to present our experience with TKA in elderly patients suffering from knee arthritis and supracondylar fractures of the femur.

## 2 Materials and methods

Ethical approval was obtained for this investigation from the Ethics Committee of Xi’an Jiaotong University Health Science Center (Ethical permit number: 2017043). From January 2009 to December 2013, 21 patients who suffered complex supracondylar femoral fractures combined with knee arthritis were treated with TKA using a stemmed femoral implant. The same observer reviewed all patients’ anteroposterior and lateral radiographs before surgery. The Arbeitsgemeinschaft für Osteosynthesefragen/Orthopaedic Trauma Association (AO/OTA) system^[16]^ was used to classify the supracondylar femoral fractures. Nine patients have typed A fracture (6 type A2 and 3 type A1), and the other 15 patients were type C fractures (9 type C1, 4 type C2, and 2 type C3) (Table 1). The operation procedure and possible complications were explained to the patients, and informed consent was all signed by the patients before the surgery.

**Table 1:**
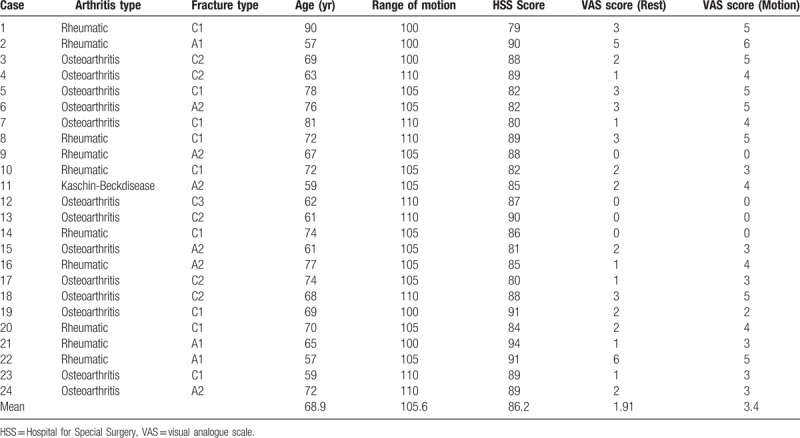
Patient characteristics at the latest follow-up; summary of the condition of patients in different categories.

All surgery was performed by the same experienced orthopedic specialist. All surgeries were performed under general anesthesia. A standard midline skin incision was used. The incision was extended until the tibial tubercle was revealed. The medial parapatellar approach was used to open the joint capsule. After the revelation of the fractures, the tibial plateau was trimmed as the reference for femoral osteotomy. Depending on different fracture situations, Kirschner wire or titanium cable was used to stabilize the fracture reduction during the surgery. The distal femoral cut was performed first, followed by a posterior condylar cut. After all femoral osteotomies were performed, a joint prosthesis with a femoral stem was implanted, and bone cement was filled into medullary cavity to stabilize the prosthesis. Additional fixation was performed on patellar cartilage surface to reach the satisfaction of the joint ROM.

After surgery, the standard antibiotic was used for 5 to 7 days under the guidelines in our hospital. The standard antibiotics we used were mezlocillin sodium, sulbactam sodium, and cefmetazole sodium. A previous study suggests that a longer period of using antibiotics can reduce the morbidity of periprosthetic joint infection.^[17]^ As most of the patients were elderly people and periprosthetic joint infection (PJI) might occur and leads to further surgery complications, we extend the application period of antibiotics up to 5 to 7 days to lower the risk of PJI. Anticoagulant was used for 2 weeks, and negative pressure drainage was planted for 2 days.

A systematic follow-up including clinical knee function, radiographic evaluation, and patients’ self-satisfaction was carried out regularly. The first evaluation was performed one-month post-surgery. Then, 2 follow-ups were requested every 3 months after the first one. Subsequently, yearly follow-up was executed after a median period of 38 months (Table 1).

For clinical evaluation, the Hospital for Special Surgery (HSS) knee scores, Visual Analog Scale (VAS) scores, and ROM were used to evaluate the recovery of knee function, including pain, ROM, function, knee flexion deformity, muscle strength, and joint stability. If a patient died during the follow-up, patient's cause of death and the knee function of the patient before death were gained from the patient's family. Patients with combined diseases affecting knee function, like hemiplegia and lumbar degenerative disease, were excluded from the study.

The anteroposterior and lateral radiography was used as the radiographic evaluation. The radiological examination was performed by standard radiographs in every follow-up. For detection of radiolucent lines, the Knee Society rating system was used.^[18]^ A radiolucent line of more than 4 mm at one place or wide distribution of a radiolucent line of more than 2 mm were regarded as a sign of prosthetic loosening.

At the latest follow-up, patients were asked for satisfactory level with the operation outcomes. Moreover, they were asked for the changes if any concerning the use of walking aids or walking distance since the surgery.

## 3 Results

**Figure 1:**
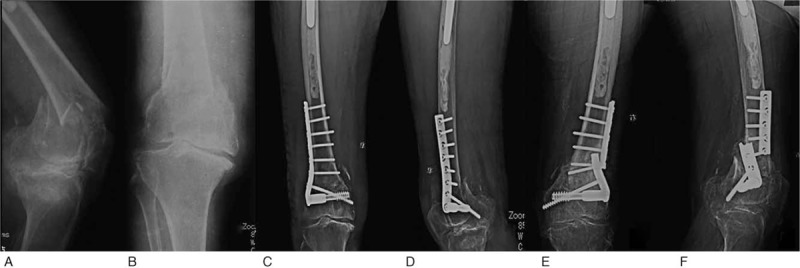
X-ray image of a female patient aged 90 years with the AO type C supracondylar femoral fracture before (A, B) and after traditional open reduction and internal-fixation (C, D). The implanted plate broke before the fracture had healed (E, F). AO = Arbeitsgemeinschaft für Osteosynthesefragen.

We present our experience with 24 cases of complex supracondylar femoral fractures in patients with knee arthritis treated with primary total knee arthroplasty with the femoral stem. The average age of the patients at operation was 68.8 years. The eldest patient was 90 years old, and 8 patients were more than 70 years old (Table 1). 2 patients had surgery for treating supracondylar femoral fractures. The percutaneous steel plate was used in both cases. One of the steel plates was broken 4 months after previous surgery (Fig. 1). Other 12 patients had no history of knee surgery. Of all the 24 patients, 4 had rheumatic knee arthritis, 9 had knee osteoarthritis, and one had Kashin–Beck disease (Table 1). The mean time of the surgery was 125 minutes, but for the re-operation patients, time of the surgery could be up to 170 minutes. The average ROM was 105.6° in the end of the follow-up period after 18 months. Overall, 17 out of 24 patients were satisfied with the outcome of the operation. The mean blood loss during the operation was 310 mL (range from 200 to 450 mL). The average of hospitalization days was 16 days (range from 14 to 20). The mean time of follow-up was 38 months (range from18 to 60 months). For all patients, the mean VAS for pain at rest was 1.9, and for motion, was 3.4. Seven patients reported the pain in the affected knee joint, 3 had pain in the knee and lower leg, and 4 did not have any pain. (Table 1). At radiographic evaluation of patients at follow-up we noted, there is no angular deformity, malunion, or shortening. Radiolucent lines on the femoral side were seen in 6 patients while they were found on the tibial side in 3 patients. There were only 3 implants with radiolucent lines of grade III (> 2 mm). Based on the radiographic and HSS score, all knee implants were stable and patients could perform (Table 2). At the final follow-up, the average ROM was 105.6°(range, 100°-110°) and the average HSS score were 86.2 (range, 79 to 94). Only 3 patients were unsatisfied with the operation outcome (Table 3).

**Table 2:**
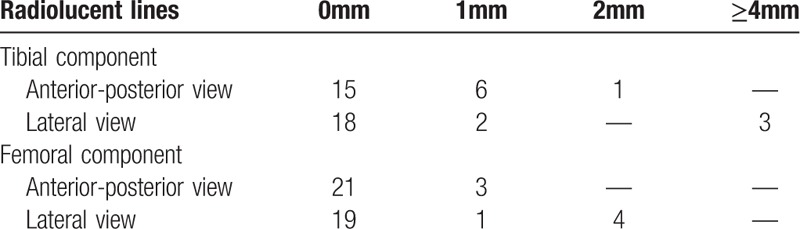
Radiographic results at the latest follow-up; summary of the outcome of surgery at follows-up.

**Table 3:**
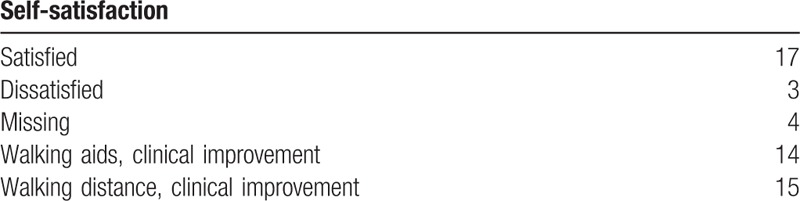
Self-satisfaction at the latest follow-up.

**Figure 2:**
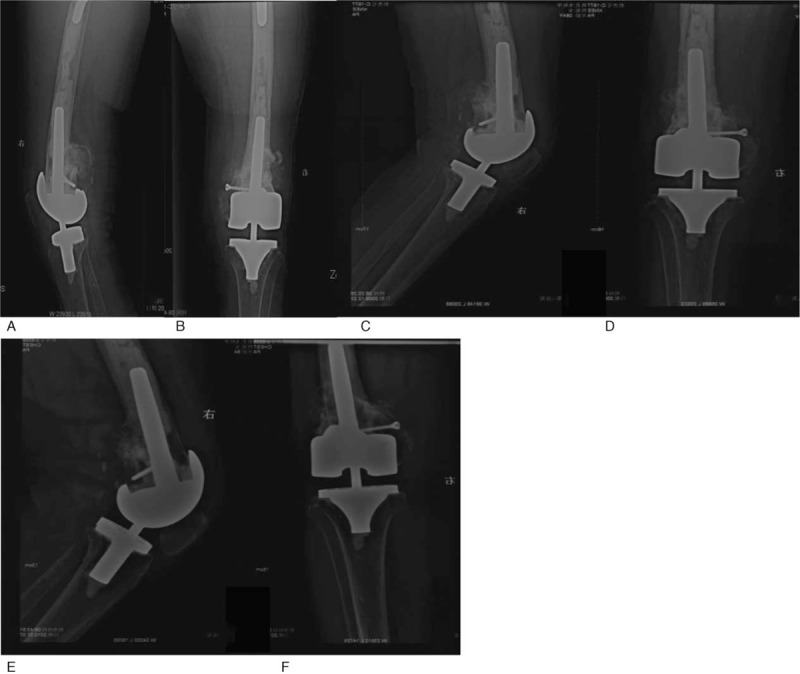
X-ray images illustrating TKA with femoral stem implantation. A hinged TKA with a femoral stem was performed on the patient described in Figure 1. The additional nail was used to stabilize the fracture. Radiographs 19 days post surgery (A, B) showed a well-maintained prosthesis. Radiographs 4 months (C, D) and one year (E, F) post-surgery suggested good knee function. TKA = total knee arthroplasty.

We first conducted this surgery for 2 patients aged 90 and 57 years and further followed up their condition for 5 years (Case No.1 and No.2 in Table 1). The female patient aged 90 years suffered from severe arthritis and osteoporosis. This patient had suffered supracondylar fractures of the femur 7 years ago and had been treated by open reduction and internal fixation of the femoral fracture (AO/OTA classification C) in another hospital (Fig. 1). Four months later, the plate broke before the fracture had healed. The patient underwent a plaster fixation and had a secondary fracture 5 months after the first treatment (Fig. 1). The patient was unable to walk for 9 months. We removed the steel plate and performed TKA with a long-stemmed component. The patient was able to exercise 19 days after surgery, and the fracture was stabilized due to the long stem. X-ray of this patient 19 days, 4 months, and 1-year post-surgery (Fig. 2) showed good condition in recovery. Sixty months after surgery, this patient was still able to walk on her own using crutches.

**Figure 3:**
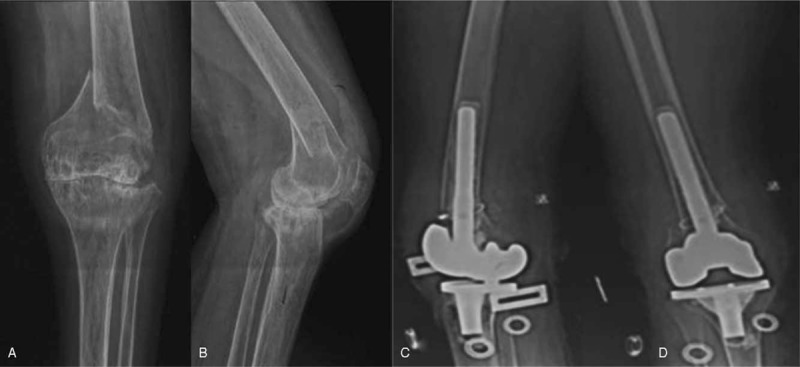
X-ray images illustrating TKA with femoral stem implantation for a patient with arthritis. Preoperative radiographs (A, B) of a female patient aged 57 years show an AO type A supracondylar femoral fracture with severe knee arthritis. A normal TKA complicated by a femoral stem was implanted in. Postoperative radiographs obtained 6 days after the operation show good alignment of prosthesis with the stem (C, D). AO = Arbeitsgemeinschaft für Osteosynthesefragen, TKA = total knee arthroplasty.

The second patient, a lady aged 57 years suffered from severe rheumatic arthritis for approximately 30 years and presented with supracondylar fractures (AO/OTA classification A) (Fig. 3A, B). After the implantation of TKA with a femoral stem, she was able to move her knee 14 days after surgery (Fig. 3C and D). Notably, both patients suffered from severe osteoporosis and comminuted fracture, and the bone defect in the fractures and both had suffered significant bone defects in the fracture zones.

After the successful trials on 2 elderly patients with severe arthritis, our treatment with 5 years’ follow-up showed adequate recovery and no further complications. Therefore, we performed this method in 22 subsequent patients, with an average of 38-months of follow-up. We summarized our results in Tables 1–3.

## 4 Discussion

The traditional way to treat displaced supracondylar fractures of the femur is open reduction, open restoration and internal-fixation.^[2,19]^ Due to knee arthritis, postoperative ankylosis and severe limitation of ROM was observed in many patients. Even if the ROM is regained, the arthritic pain is still unbearable. The 2-step TKA is suitable to treat this kind of fracture. However, elderly patients often need more time to recover from a major operation. The second surgery introduces an additional risk of anesthetic complications, postoperative infections, and other complications. Moreover, since osteoporosis is commonly observed in elderly patients, additional surgery may increase the instability of the fracture, or even cause secondary fractures. Therefore, multiple alternative treatments had been reported to improve this 2-step procedure. Among them, one-step TKA with a femoral stem is an efficient way to treat this kind of fractures. During this procedure, knee arthritis and supracondylar fracture can be treated simultaneously. In this way, the arthritis pain is relieved and knee function can be improved greatly. Moreover, the femoral stem and titanium cable in the surgery can stabilize the complex fracture.

In our study, all patients were elderly female patients with supracondylar fractures. All patients were diagnosed with osteoporosis. The articular surface was involved in most of the patients. All patients had been suffering from severe arthritic pain and were eager to restore knee function and relieve the pain at the time of fracture reduction. A femoral stem was used in every patient because of the complex and unstable fractures. For patients suffering from knee arthritis, mobilization in the early stage is a crucial step to maintain knee functions and to avoid medical complications of prolonged bed-bound state. After surgery, most of our patients were allowed to mobilize at an early stage without needing ancillary splintage. In a rather short in-patient stay, the arthralgia was significantly reduced, and the average ROM was 74.3° at the time of hospital discharge (range: 70°–83°). In our TKA procedure, the mean time of hospitalization is 16 days while the statics shows that the mean hospital stay for patients undergoing open reduction and internal was 31 days.^[5,20,21]^

Our study and technique have some limitations. First of all, the number of patients is relatively small due to the specific requirements of patient recruitment in this study. Elderly patients are usually more concerned about new techniques and are inconvenient to come to hospitals for repeat follow-up visits, which makes it difficult to recruit a large number of patients with high potential for long-term studies. Second, there was no control group with traditional surgery for comparisons in parallel, regarding the complexity and time of the procedures, the therapeutic outcomes, and long-term follow-ups. Third, as the patients involved in this study were elderly residents, a 5-year follow-up might be still insufficient for integrative insights regarding the outcomes and side effects of this procedure. Therefore, a longer period of follow-up and better involvement of patients after surgery is needed to test the efficiency of this type of treatment. This 1-step procedure requires a higher level of surgical skills. Because of the complex fracture in combination with arthritis and the loss of bone fragments, it is challenging for the surgeons to elaborate the optimal plan for treatment. Concerning the required surgical practice and experience, and the possible side effects, such as the probability of infections due to longer surgery time, it is more difficult to apply this 1-step surgery as treatment in primary healthcare centers.

Although there are some concerns that peer clinicians and surgeons need to further address, our study achieved a highly satisfactory outcome. Our imaging results and functional follow-up analyses revealed positive outcomes. In our mid-term follow-up no significant complication was reported. One patient died of disease unrelated to knee arthritis, the remaining patients are alive and report an improved knee function and reported a great reduction of arthritis pain. Recently, other case studies using this 1-step surgery also reported similar positive outcomes with 8 and 5 patients.^[22,23]^ Therefore, we believe that TKA with a femoral stem is a reasonable alternative for the treatment of supracondylar femoral fracture in patients suffering from coexisting arthritis of the knee.

## 5 Conclusions

Based on the clinical and radiographic results, the 1-step TKA with femoral stem can be considered as an alternative for the treatment of complex supracondylar femoral fractures in elderly patients suffering from massive knee arthritis.

## Acknowledgment

The authors thank Dr Jun Li for scientific discussion, Dr Xiaofei Li from Karolinska Institute, Sweden for scientific discussion and manuscript editing.

## Author contributions

Kunzheng Wang conceptualized the project.

Wei Wang and Kun Yang designed and performed experiments.

Wei Wang, Kun Yang, Pei Yang, Dandan Song, Chunsheng Wang, Jinhui Song, and Xiaohui Li performed the analyses.

Kun Yang and Kunzheng Wang wrote the paper.

**Conceptualization:** Kunzheng Wang.

**Data curation:** Wei Wang, Kun Yang, Pei Yang, Dandan Song, Chunsheng Wang, Jinhui Song, Xiaohui Li.

**Formal analysis:** Wei Wang, Kun Yang, Pei Yang, Dandan Song, Chunsheng Wang, Jinhui Song, Xiaohui Li.

**Funding acquisition:** Kunzheng Wang.

**Investigation:** Kunzheng Wang.

**Methodology:** Kunzheng Wang.

**Project administration:** Kunzheng Wang.

**Resources:** Kunzheng Wang.

**Supervision:** Kunzheng Wang.

**Validation:** Kunzheng Wang.

**Writing – original draft:** Kunzheng Wang.

**Writing – review & editing:** Kunzheng Wang.
